# Testosterone Levels in Transgender Women Undergoing Gender-Affirming Hormone Therapy

**DOI:** 10.7759/cureus.83365

**Published:** 2025-05-02

**Authors:** Emily W Miro, Katherine Rizzone, Kory Ford, Tiffany F Ho, Erika Sullivan, Bayarmaa Mark, Masaru Teramoto, Dan Cushman

**Affiliations:** 1 Department of Family and Preventive Medicine, University of Utah Health, Salt Lake City, USA; 2 Department of Orthopedics, University of Rochester, Rochester, USA; 3 Department of Physical Medicine and Rehabilitation, University of Utah Health, Salt Lake City, USA; 4 Huntsman Cancer Institute, University of Utah Health, Salt Lake City, USA; 5 Department of Orthopedics, University of Utah Health, Salt Lake City, USA

**Keywords:** gender-affirming hormone therapy, gender identity, sports, testosterone, transgender

## Abstract

The participation of transgender women in women’s sports is a topic rife with controversy and lacks a unified set of guidelines. Testosterone thresholds vary widely between organizations, ranging from less than 2.5 nmol/L to less than 10 nmol/L during a pre-competition period of 12 to 24 months. Little is known about how quickly and to what degree testosterone is suppressed below the thresholds required for participation in women’s sports after the initiation of gender-affirming hormone therapy (GAHT). This study examined trends in testosterone levels among transgender women undergoing the current standard of care GAHT and compared these values to existing guidelines for participation in women’s sports. After 12 months on GAHT, the median testosterone level was 0.52 nmol/L (95% CI: 0.47-0.73), and the mean was 3.39 nmol/L (95% CI: 2.63-4.15) (N=261). After 24 months, the median testosterone level was 0.43 nmol/L (95% CI: 0.35-0.66), and the mean was 3.90 nmol/L (95% CI: 2.51-5.29) (N=112). These results suggest substantial variability in testosterone levels among transgender women on GAHT. These data may inform the typical course of testosterone suppression under the current standard of care and serve as a reference for future athlete-specific studies. Further research is needed to better understand both the extent of testosterone suppression across different GAHT regimens and the duration required to meet eligibility guidelines for participation in women’s sports.

## Introduction

The participation of transgender women in women’s sports is a topic rife with controversy [1,2]​. The International Olympic Committee (IOC) published guidelines in 2015 stating that testosterone values among transgender women must be <10 nmol/L for at least 12 months prior to an athlete’s first competition [3]​. In 2021, the IOC’s stance changed to deferring to each sport’s national governing body to set guidelines for participating in their sport [4]. Without a unifying set of guidelines, organizations have established their policies for participation [5]. Many have utilized objective measures, such as serum testosterone values, to guide eligibility criteria for transgender women in women’s sports. Testosterone levels vary widely between individuals, ranging from less than 2.5 nmol/L to less than 10 nmol/L, during a pre-competition period of 12-24 months [6-10].

Little is known regarding how quickly testosterone is suppressed below the threshold required for participation in women’s sport after initiation of gender-affirming hormone therapy (GAHT). Additionally, data is lacking that shows the degree to which specific regimens of GAHT achieve the defined testosterone thresholds in sport. This study aimed to elucidate trends in testosterone levels among transgender women on GAHT.

## Materials and methods

Participants and procedures

Our retrospective cohort study examined clinical data from transgender women 18 and older who were seen at one academic medical system between January 2013 and April 2023. The Institutional Review Board of the University of Utah approved the study (approval number: 00161271, approval date: December 20, 2022). Waiver of consent and authorization was approved for this study. For this study, transgender women were defined as those who had at least one clinical encounter that billed specific International Classification of Diseases (ICD)-9 or ICD-10 diagnosis codes associated with gender dysphoria/transgender status (Table 1) [11,12] and documented prescriptions of estradiol and spironolactone for GAHT. Participation in sport was not an inclusion criterion of this study, as this information was not available to the authors. Protocols for initiating and titrating GAHT medications, as well as laboratory monitoring, at this institution (Tables 2-3) mirror the current standards of care recommended by the Endocrine Society and the World Professional Association for Transgender Health [13,14]. An index date was defined for each patient as the date at which the first prescription for GAHT was provided. Further details of the dataset creation were previously published by Ho et al. [15]. Serum testosterone values, previously drawn as part of routine GAHT monitoring, were analyzed.

**Table 1:** ICD-9 and ICD-10 diagnostic codes utilized to identify transgender women for cohort ICD: International Classification of Diseases [11,12]

ICD-9 diagnostic code	ICD-10 diagnostic code
302.50: trans-sexualism with unspecified sexual history (aka “trans-sexualism not otherwise specified”)	F64.0: transsexualism
302.51: trans-sexualism with asexual history	F64.1: dual role transvestism
302.52: trans-sexualism with homosexual history	F64.2: gender identity disorder of childhood
302.53: trans-sexualism with heterosexual history	F64.8: other gender identity disorders
302.60: gender identity disorder in children	F64.9: gender identity disorder, unspecified
302.85: gender identity disorder in adolescents or adults	Z87.890: personal history of sex reassignment

**Table 2:** Institutional recommendation for dosing of feminizing GAHT Institutional recommendation for dosing of feminizing GAHT, mirroring standard of care protocols for initiation and titration of GAHT medications recommended by the Endocrine Society and the World Professional Association for Transgender Health, taken from the University of California San Francisco Gender Affirming Health Program. GAHT: gender-affirming hormone therapy

Timing	Medication and recommendations
Initial dose	Estradiol:
	Oral estradiol: 2 mg by mouth daily
	Injectable estradiol valerate (20mg/mL): 1-2 mg weekly
	Transdermal estradiol: 0.1 mg twice weekly
	Spironolactone: 25-50 mg daily
After one month	Estradiol:
	Oral estradiol: increase estradiol to 2 mg by mouth twice daily
	Injectable estradiol valerate: increase by 1 mg increments
	Transdermal estradiol: increase by 0.1 mg increments
	Spironolactone: increase by 50 mg increments to 200 mg by mouth daily
Three months after starting hormones	Check labs. If labs are not within the desired range (estradiol 100-200 pg/mL and testosterone <50 ng/dL):
	Increase oral estradiol to 5 or 6 mg total daily dosing (typically 2-3 mg AM/3 mg PM)
	Increase spironolactone to the maximum dose of 400 mg daily
Every three months	Check labs. If labs are not in the desired range:
	Increase oral estradiol typically to a max of 6mg total daily dose
	Increase spironolactone to the maximum dose of 400 mg daily

**Table 3:** Institutional recommendation for laboratory monitoring of feminizing GAHT Institutional recommendation for laboratory monitoring of feminizing GAHT, mirroring standard of care protocols for initiation and titration of GAHT medications recommended by the Endocrine Society and the World Professional Association for Transgender Health, taken from the University of California San Francisco Gender Affirming Health Program. CMP: comprehensive metabolic panel, GAHT: gender-affirming hormone therapy

Timing	Laboratory tests
Baseline	Basic metabolic panel or CMP if using spironolactone
First year (or when making dose adjustment)	Estradiol
	Total testosterone
	CMP
Semi-annually or annually for years >1	Estradiol
	Total testosterone
	CMP

Statistical analyses

Mean and median testosterone values at 12 and 24 months after initial prescription for GAHT and proportions of testosterone values over 5 nmol/L and 2.5 nmol/L were calculated. These time points and serum values were chosen because they are commonly used to guide eligibility criteria for participation in sport [6-10]. A 95% confidence interval was also computed for the means, medians (calculated using bootstrapping with 1,000 replications) [16-18], and proportions (calculated based on Wilson confidence intervals) [19] above.

## Results

**Figure 1:**
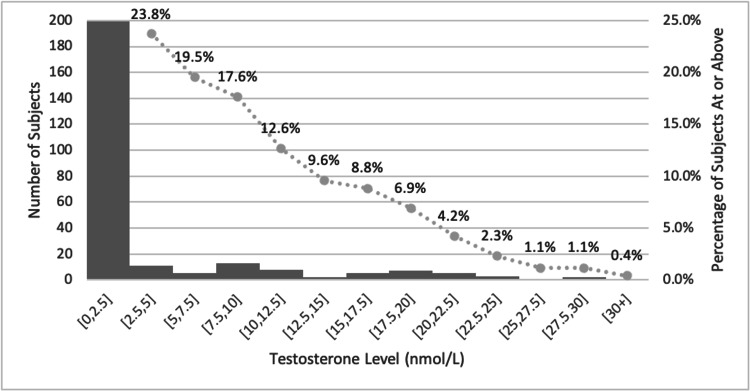
Histogram of testosterone levels (nmol/L) for all subjects at 12 months The x-axis lists the ranges of each testosterone level for each bin. The left y-axis refers to the bar graph and indicates the number of subjects in each bin. The right y-axis refers to the line graph, representing the percentage of subjects with a testosterone level at or higher than that bin. For example, 23.8% of subjects have a testosterone level of 2.5 nmol/L or higher.

A total of 261 patients met study criteria at 12 months. The mean age among this group was 28 years. After 12 months on GAHT, patients demonstrated a median testosterone of 0.52 nmol/L (95% CI: 0.47-0.73) and a mean testosterone of 3.39 nmol/L (95% CI: 2.63-4.15) (Figure 1). After 24 months, 112 patients met study criteria. The mean age of this group was 30 years. The median testosterone level among this group was 0.43 (95% CI: 0.35-0.66), and the mean was 3.90 nmol/L (95% CI: 2.51-5.29). As shown in Figure 1, at 12 months, 19.5% (95% CI: 15.2-24.8) of patients had testosterone values higher than the 5 nmol/L threshold, and 23.8% (95% CI: 19.0-29.3) had values above the 2.5 nmol/L threshold. At 24 months, 22.3% (95% CI: 15.6-30.9) and 25.0% (95% CI: 17.9-33.8) were above the respective thresholds.

## Discussion

Many organizations have testosterone-based policies informing the participation of transgender women in women’s sports. Policies vary in testosterone threshold and length of documented testosterone suppression prior to participation. Testosterone levels of less than 10, 5, or 2.5 nmol/L are required by many organizations for varying periods prior to participation (Table 4) [6-10]. However, there is a paucity of data supporting the use of these specific thresholds. Moreover, recent publications suggest that testosterone suppression post-puberty does not negate the male performance advantage; persistent biomechanical advantages are seen in transgender women, namely muscle mass and explosive strength [1,20,21].

**Table 4:** Select published guidelines for participation in sports by organization* * Information accurate as of January 28, 2025

Organization	Country/region	Testosterone threshold for participation	Interval for required testing
World Aquatics [6]	International	2.5 nmol/L	In androgen-sensitive athletes, male puberty must be suppressed beginning at Tanner stage 2 or by age 12, whichever is later, AND have continuously maintained testosterone below 2.5 nmol/L.
World Rowing [7]	International	2.5 nmol/L	At least 24 months prior to the athlete’s first competition.
Union Cycliste Internationale (UCI) Cycling [8]	International	2.5 nmol/L	In androgen-sensitive athletes, male puberty must be suppressed beginning at Tanner stage 2 or by age 12, whichever is later, AND have continuously maintained testosterone below 2.5 nmol/L.
International Tennis Federation (ITF) [9]	International	5 nmol/L	At least 12 months prior to the athlete’s first competition.
USA Wrestling [10]	United States	10 nmol/L	At least 12 months prior to the athlete’s first competition.

Many studies exist that review testosterone values among cisgender women. One meta-analysis aggregating nine studies demonstrated a 95% confidence interval for testosterone of 0-1.7 nmol/L [22]. The same meta-analysis showed that among women with polycystic ovary syndrome, a common condition associated with increased circulating testosterone levels, the mean testosterone level was 1.69 nmol/L, with an upper limit of 3.13 nmol/L (95% CI, one-sided) or 4.77 nmol/L (99.99% CI, one-sided) [22].

Current Endocrine Society guidelines define the goal testosterone for transgender women as <1.73 nmol/L [14]. Nonetheless, published data on testosterone levels among transgender women vary. One previous study of 40 participants reported mean testosterone levels of 0.52 nmol/L and 0.59 nmol/L after 12 and 24 months of GAHT, respectively. Another study of 275 participants reported mean levels of 0.40 nmol/L after both 12 months and two to four years on GAHT [23,24]. Notably, participation in sports was not a criterion for eligibility in either study.

Our study found that transgender women had a mean testosterone of 3.39 nmol/L after 12 months of GAHT and 3.90 nmol/L after 24 months on GAHT. These values are significantly higher than the median values of 0.52 nmol/L and 0.43 nmol/L at 12 and 24 months on GAHT, respectively. The positively skewed data in our study shows that while many patients achieve significant suppression of testosterone values on standard doses of GAHT, this regimen may not be adequate for all patients. Additionally, this retrospective data likely describes testosterone levels of transgender women with typical use of GAHT versus perfect use in those mentioned above, highly standardized, prospective studies.

Collectively, these data show wide variability in testosterone levels among transgender women on the current standard of care regimens of GAHT. This may be related to variability in patient compliance with treatment regimens, patient population, or laboratory testing.

Limitations 

Our study has several limitations. The authors acknowledge that many individuals taking estradiol-based GAHT do not identify as transgender women. For this study, transgender women were defined as those who met the combined criteria of an ICD-9 or ICD-10 diagnosis associated with gender dysphoria/transgender status and prescriptions for GAHT. Although these patients had documented prescriptions for estradiol and spironolactone, it is possible that these medications were not taken as prescribed. Nonadherence to GAHT may affect testosterone values obtained at the 12- and 24-month time points. This may highlight the typical use versus the ideal use of hormones in prior studies. Patients were not excluded if they had received previous gender-affirming surgeries. Our final cohort of 112 patients at 24 months was significantly smaller than the initial cohort of 261 patients at 12 months, as many were likely lost to follow-up or had transitioned their care to another institution. Finally, participation in sport was not an inclusion criterion of this study, as this data was unavailable to the authors.

## Conclusions

While many organizations have included testosterone thresholds in their guidelines for participation of transgender women in women’s sports, limited studies exist describing typical trends in testosterone among transgender women on GAHT. In our sample, almost one quarter of patients did not achieve testosterone suppression below the thresholds of 2.5 and 5 nmol/L proposed by many organizations after one and two years on the current standard of care GAHT guidelines. These data may be used to inform the typical history of testosterone levels on the current standard of care GAHT therapy for future athlete-specific studies to reference. Further understanding of both the degree of testosterone suppression on different regimens of GAHT and the duration of GAHT required to meet guidelines for participation in women’s sport is needed to inform guidelines for participation of transgender women in women’s sport.
